# Red wine consumption increases antioxidant status and decreases oxidative stress in the circulation of both young and old humans

**DOI:** 10.1186/1475-2891-6-27

**Published:** 2007-09-24

**Authors:** Michelle Micallef, Louise Lexis, Paul Lewandowski

**Affiliations:** 1 School of Biomedical Sciences, University of Newcastle, New South Wales, Australia; 2 School of Biomedical Sciences, Victoria University, Victoria, Australia; 3 School of Medicine Deakin University, Victoria, Australia

## Abstract

**Background:**

Red wine contains a naturally rich source of antioxidants, which may protect the body from oxidative stress, a determinant of age-related disease. The current study set out to determine the in vivo effects of moderate red wine consumption on antioxidant status and oxidative stress in the circulation.

**Methods:**

20 young (18–30 yrs) and 20 older (≥ 50 yrs) volunteers were recruited. Each age group was randomly divided into treatment subjects who consumed 400 mL/day of red wine for two weeks, or control subjects who abstained from alcohol for two weeks, after which they crossed over into the other group. Blood samples were collected before and after red wine consumption and were used for analysis of whole blood glutathione (GSH), plasma malondialdehyde (MDA) and serum total antioxidant status.

**Results:**

Results from this study show consumption of red wine induced significant increases in plasma total antioxidant status (P < 0.03), and significant decreases in plasma MDA (P < 0.001) and GSH (P < 0.004) in young and old subjects. The results show that the consumption of 400 mL/day of red wine for two weeks, significantly increases antioxidant status and decreases oxidative stress in the circulation

**Conclusion:**

It may be implied from this data that red wine provides general oxidative protection and to lipid systems in circulation via the increase in antioxidant status.

## Background

Efforts to define the role of nutrition in health have captured researcher's interest in antioxidants and their capacity to protect the body from damage induced by oxidative stress. Extensive research has demonstrated the protective properties of antioxidants, which scavenge reactive oxygen species (ROS) and their precursors, as well as up-regulate enzymes involved in the repair of cellular damage [1]. Red wine contains a rich source of a large number of antioxidants, namely the phenolic acids and polyphenols, which provide it with its protective redox potential [2,3].

Epidemiological studies have shown that despite the high intake of saturated fatty acids within the diets of some populations, a reduced mortality rate from cardiovascular disease is attributed to the high consumption of red wine, independent of its alcohol content, the 'French Paradox' [4]. Studies also indicate that sub-populations already at a high risk of coronary heart disease (CHD) (i.e. elderly) may potentially experience a greater beneficial effect from moderate wine consumption [5]. Moderate consumption of red wine has also been shown to retard or slow the plasma clearance of high density lipoproteins (HDL), a negative risk factor for the development of cardio vascular disease (CVD). In doing so, a positive correlation between HDL particles and moderate red wine intake becomes evident [6]. Furthermore, the incubation of low density lipoproteins LDL) in varying concentrations of red and white wine showed a 50% decline in oxidation at concentrations of 0.04 and 0.7 mg/ethanol/mL respectively, up to a concentration of 1.0 mg/mL. These results indicate that red wine inhibits cell mediated LDL oxidation more efficiently then white wine and at much lower concentrations.

To investigate further, the relationship between red wine consumption and oxidative damage in humans has been studied by Greenrod and Fenech [7], in a series of in vitro and ex vivo study designs. They demonstrated a strong (> 70%) reduction in H2O2 induced genetic damage after 1-hour post consumption of 300 mL of red wine. These findings are also supported by a similar study by Szeto and Benzie [8], showing that DNA damage was significantly reduced in a H2O2 challenge, with treatment of caffeic acid, a polyphenol found in red wine.

Oxidative damage to a range of biomolecules is of particular interest to researchers. The tripeptide glutathione (GSH) functions as an antioxidant, which scavenges free radical species in circulation. GSH is oxidized as the enzyme glutathione peroxidase catalyzes the degradation of H2O2 [9]. Increasing evidence demonstrates GSH plays an integral role in the protection against oxidative stress in the circulation due to its ability to facilitate the recycling of oxidized α-tocopherol and ascorbic acid, two important antioxidants in the circulation and is widely used as a biomarker of circulating antioxidant levels [10]. Within plasma fatty acid residues of phospholipids and LDL, are extremely susceptible to oxidative damage by free radical intermediates resulting in oxidized fatty acids and peroxidation byproducts, such as conjugated diennes (CD) and malondialdehyde (MDA) derivatives [11]. MDA appears to be one of the most toxic and mutagenic aldehydes generated by lipid peroxidation of polyunsaturated fatty acids of cell membranes [12]. It is also a popular measurement used to quantify the effects of radical damage to cellular lipids [13].

A large body of evidence which indicates that free radical production can directly or indirectly play a major role in cellular processes implicated in atherosclerosis and CVD, [14]. Therefore the aim of this study were firstly to understand how moderate red wine consumption (400 ml/day) for two weeks effected circulating lipids, antioxidant level and total antioxidant capacity in the circulation and secondly assess the differences in bioefficacy of red wine in young and older populations.

## Methods

### Recruitment of volunteers

**Figure 1:**
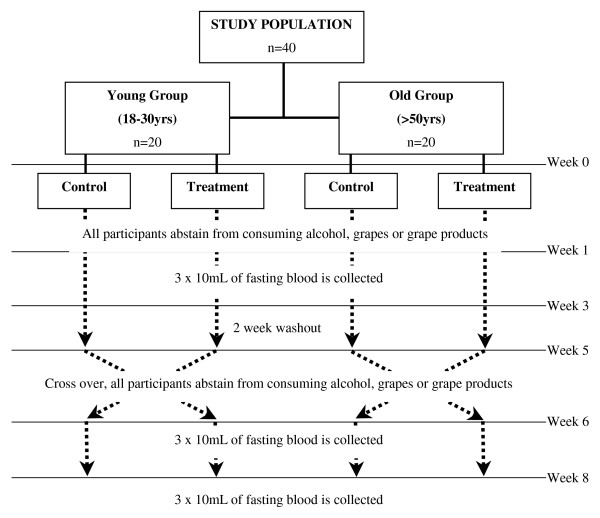
**Outline of study**. Volunteers consumed 400 mL/day of red wine for two weeks or abstained from consuming alcohol, grapes or grape products for two weeks and crossed over into the other group after a two week washout.

This study protocol was approved by the Human Research Ethics Committee of Victoria University (HRETH.SET 15/05). Forty volunteers were selected based upon their responses to a general health questionnaire and after giving written informed consent. Those who were taking any anti-coagulant or anti-inflammatory medications or had a history of cardiovascular or liver disease were excluded. Two age groups were selected, these were 20 volunteers aged between 18–30 years old (young group) and 20 volunteers aged older then 50 years old (older group). Volunteers were randomly assigned to begin in the red wine or control group within their respective age group (Figure 1).

### Intervention design

Prior to drinking the red wine or control period volunteers were asked to abstain from consuming any alcohol, grapes or grape products for one week. After this one week lead in subjects had three 10 mL tubes of fasting blood collected via venipuncture to determine baseline measures of MDA, GSH, and total antioxidant capacity and BMI (kg/m^2^) calculated, after which they began the red wine or control period. During the red wine period participants consumed 400 mL of red wine each day (Cabernet Sauvignon) over a period of two consecutive weeks and abstained from other alcohol, grapes or grape products. A placebo such as alcohol free wine was not used due to difficulties in matching the flavour and mouth feel of the red wine used. Instead a crossover design was used whereby after completing either the red wine or control period volunteers were given a two week washout period before crossing over into the other group. During the control period volunteers abstained from consuming any source of alcohol, grapes or grape products for two weeks. Three 10 mL tubes of fasting blood were again collected after the treatment or control phase (see Figure 1). Participants were also encouraged to maintain their usual diet and exercise habits throughout the entire study phase which was monitored by participants keeping a food and activity diary before and during the study. There were no specific instructions given to avoid foods containing large amounts of phenolic compounds, other than abstain from consuming any alcohol, grapes or grape products as previously described.

### Wine supplementation

The red wine used throughout this study was a Cabernet Sauvignon, supplied as a cask wine to prevent the oxidation of the wine. This style was chosen since it is known to be palatable to most people and to the volunteers in the study. Participants consumed the wine at any time during the day, however, it was suggested that they do so at a time when they would normally consume alcohol (e.g. with an evening meal). Importantly, during the period of supplementation participants were asked to refrain from consuming any other sources of alcohol, grapes or grape products.

### Wine composition

The concentrations of total anthocyanins, degree of anthocyanin ionisation, total phenolic compounds, red wine colour (density and hue) and two indices providing a measure of polymerisation of monomeric forms (Chemical age index #1 and #2) were determined by spectrophotometric methods [15,16]. Determination of the concentration of free and bound sulphur dioxide in the wine was made using the method of Rankine and Pocock [17]. Alcohol content was provided by the wine producer. The composition of the wine used in this study was analysed can be seen in Table 1. All components of the wine used in this study, except for red wine colour – hue and free sulfur dioxide, were slightly higher than the red wine used in a study by Greenrod et al[18].

**Table 1:** Composition of wine used in study

**Compounds**	**Amount**
Total Anthocyanins	129 mg/L
Degree of ionisation of anthocyanins	24.85 %
Degree of ionisation of anthocyanins-abolishing effect of sulphur dioxide	31.56 %
Total phenolics	40 a.u.
Total polyphenols^a^	1.558 g/L
Red wine colour – density	8.4 a.u.
Red wine colour – hue	0.83 a.u.
Chemical age index #1	0.59 a.u.
Chemical age index #2	0.26 a.u.
Free sulfur dioxide, excluding anthocyanin bound sulphur dioxide	1.04 mg/L
Alcohol	13.0 %

^a ^Epicatechin equivalents.

a.u. Absorbance units.

### Analyses of glutathione

Glutathione was measured as it is an important antioxidant in the circulation using a commercially available colorimetric kit (Northwest Life Sciences) based on the method of Teitze [19] following the manufactures instructions. Blood was collected via venipuncture using EDTA coated tubes and stored at 4°C. Whole blood samples were then deproteinated mixing aliquots with 100 ul of cold 5% metaphosphoric acid followed by centrifugation at 1500 × g for 5 min, the supernatant was then removed and stored at -20°C awaiting further analysis. All samples were then assayed for reduced GSH as a batch. This involved mixing 50 μL of calibrators or samples with 50 μL DTNB reagent and 50 μL glutathione reductase reagent in the wells of microplate. This reaction mix was then incubated at ambient temperature for 3 min after which 50 μL NADPH reagent was added to all wells and absorbance values read at 405 nm with data collected at 15 sec intervals for 3 min. Absorbance values were then plotted as a function of time for each calibrator and sample. A calibration curve was then constructed by plotting the ^Δ ^A405/min for each calibrator as a function of the GSH concentration and the equation for the calibration curve was then used to calculate the concentration of GSH in all samples.

### Analyses of malondialdehyde

Plasma malondialdehyde was as a marker of lipid peroxidation using a commercially available colorimetric kit (Northwest Life Sciences) following the manufactures instructions. Blood was collected via venipuncture using EDTA coated tubes, stored at 4°C and plasma separated within 2 hrs by centrifugation at 3000 × g for 10 minutes at room temperature. Plasma samples were then stored at -20°C awaiting further analysis. All samples were then assayed for MDA as a batch. This involved mixing 250 μL calibrator or sample with 10 μL of Butylated hydroxytoluene reagent, 250 μL Phosphoric acid reagent and 250 μL 2-Thiobarbituric acid reagent. This reaction mix was then incubated at 60°C for 60 min followed by centrifugation at 10000 × g for 3 min. Absorbance of calibrators and samples was then read at 532 nm in a spectrophotometer (Biorad). Absorbance values for calibrators were then used to construct a calibration curve and the equation for calibration curve was then used to calculate the concentration of MDA in all samples.

### Analyses of total antioxidant status

Serum total antioxidant status (TAS) was determined for a quantitative assessment of in vivo antioxidant status using a commercially available kit (Randox) based on the trolox equivalent antioxidant capacity method of Miller [20] following the manufactures instructions. Blood was collected via venipuncture using serum separator tubes, stored at 4°C and serum separated within 2 hrs. Serum samples were then stored at -20°C awaiting further analysis. All samples were then assayed for TAS as a batch. This involved mixing 20 μL calibrator (6-hydroxy-2,5,7,8-tetramethylchroman-2-carboxylic acid 1.79 mmol/L) or sample with 1 ml of chromogen (metmyoglobin 6.1 μmol/L, ABTS 610 μmol/L) and incubating at 37°C for 3 min. Initial absorbance was then read at 600 nm in a spectrophotometer (Biorad). After which 200 μL of substrate (hydrogen peroxide 250 μmol/L) was added to calibrator and sample and incubated at 37°C for 3 min. Final absorbance was then read at 600 nm. The change in absorbance value for samples relative to the change in absorbance of the calibrator was then to calculate the TAS in all samples. The total antioxidant status of the red wine (Cabernet Sauvignon) used in this study was also measured using the same assay.

### Analyses of serum glucose and plasma lipids

Serum glucose was determined using a commercial glucose oxidase reagent and standard (Thermo Electron Corporation). This involved mixing 3 μL of calibrator or sample with 450 μL of glucose oxidase reagent and incubating at 37°C for 5 min. Absorbance of calibrators and samples was then read at 500 nm in a spectrophotometer (Biorad). The absorbance value of samples relative to the absorbance of the calibrator was then to calculate the glucose level in all samples. Plasma triglycerides were determined using commercially available colorimetric kit (Thermo Electron Corporation). This involved mixing 6 μL of calibrator or sample with 600 μL of triglyceride reagent and incubating at 37°C for 3 min. Absorbance of calibrators and samples was then read at 500 nm in a spectrophotometer (Biorad). The absorbance value of samples relative to the absorbance of the calibrator was then to calculate the triglyceride level in all samples. Total cholesterol was determined using commercially available colorimetric kit (Thermo Electron Corporation). This involved mixing 6 μL of calibrator or sample with 600 μL of cholesterol reagent and incubating at 37°C for 3 min. Absorbance of calibrators and samples was then read at 500 nm in a spectrophotometer (Biorad). The absorbance value of samples relative to the absorbance of the calibrator was then to calculate the cholesterol level in all samples. HDL cholesterol was determined using commercially available colorimetric kit (Thermo Electron Corporation). This involved mixing 4 μL of calibrator or sample with 300 μL of HDL reagent 1 and incubating at 37°C for 5 min. After which 100 μL of HDL reagent 2 was added to calibrator and sample and incubated at 37°C for 3 min. Absorbance of calibrators and samples was then read at 600 nm in a spectrophotometer (Biorad). The absorbance value of samples relative to the absorbance of the calibrator was then to calculate the triglyceride level in all samples. LDL cholesterol, a risk factor for cardiovascular disease, was calculated by subtracting HDL cholesterol values, a negative risk factor for cardiovascular disease, from total cholesterol.

### Statistical analysis

Statistical analysis was performed using the SPSS statistical package (version 12.0, SPSS Inc.). All data were distributed normally and expressed as mean ± standard error of the mean (SEM). Data from young and older individuals were analyzed using a three way ANOVA to determine the effect of wine consumption within the young or old group, any difference between young and old groups and any difference between pre samples with the young or old group. Due to the cross over design of the study any difference between are not included in the analysis s the primary focus of the research was to determine the effect of red wine consumption. In all cases a P value of < 0.05 was considered statistically significant.

## Results

**Figure 2:**
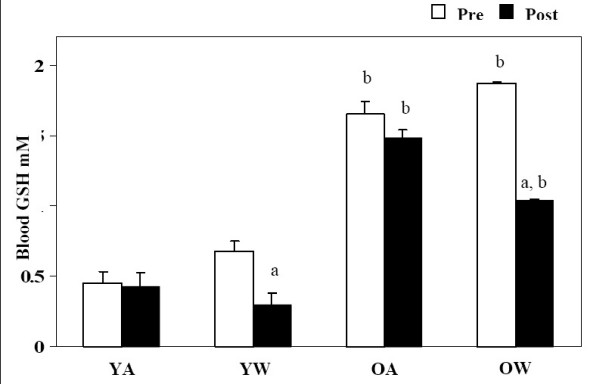
**Blood glutathione**. Effect of red wine consumption on blood GSH concentrations from young (18–30 yrs) and older (≥ 50 yrs) volunteers. Bars represent mean ± SEM of blood GSH values pre and post two weeks of red wine consumption or abstinence. YA, young abstinence; YW, young wine; OA, older abstinence; OW older wine. ^a ^indicates a significant change with wine P < 0.05. ^b ^indicates a significant difference between young and old, P < 0.05.

Whole blood glutathione was measured as it is an important circulating antioxidant. Before and after red wine consumption GSH levels were elevated in older volunteers compared to young volunteers (P < 0.001, Figure 2). Despite this difference between young and old volunteers consumption of red wine had the same effect with both the young and old groups causing significant reductions in GSH levels after red wine consumption, young with wine (P = 0.004) and older with wine periods (P < 0.001, Figure 2). No significant changes in GSH level were observed in young and older groups without red wine.

**Figure 3:**
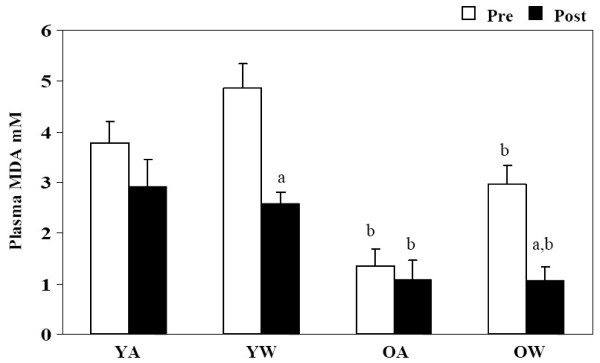
**Plasma malondialdehyde**. Effect of red wine consumption on plasma MDA concentrations from young (18–30 yrs) and older (≥ 50 yrs) volunteers. Bars represent mean ± SEM of plasma MDA values pre and post two weeks of red wine consumption or abstinence. YA, young abstinence; YW, young wine; OA, older abstinence; OW older wine. ^a ^indicates a significant change with wine P < 0.05. ^b ^indicates a significant difference between young and old, P < 0.05.

Plasma malondialdehyde was measured as a biomarker of lipid peroxidation. Before and after red wine consumption MDA levels were reduced in older volunteers compared to young volunteers (P < 0.05, Figure 3). Despite this difference between young and old volunteers consumption of red wine had the same effect within both the young and old group causing significant reductions in MDA levels after red wine consumption, young with wine (P < 0.001) and older with wine periods (P < 0.001, Figure 3). No significant changes in MDA level were observed in young and older groups without red wine.

**Figure 4:**
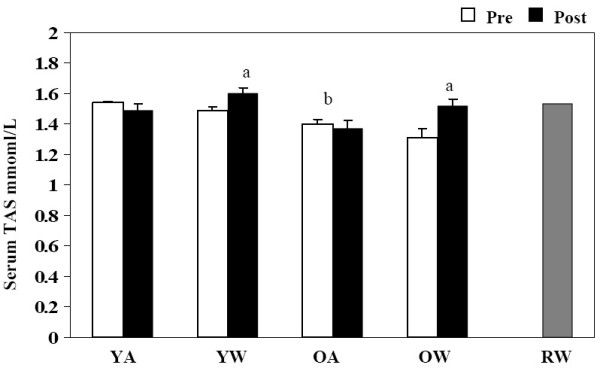
**Serum total antioxidant status**. Effect of red wine consumption on serum TAS from young (18–30 yrs) and older (≥ 50 yrs) volunteers. Bars represent mean ± SEM of serum TAS pre and post two weeks of red wine consumption or abstinence. YA, young abstinence; YW, young wine; OA, older abstinence; OW older wine; RW red wine ^a ^indicates a significant change with wine P < 0.05. ^b ^indicates a significant difference between young and old, P < 0.05.

Serum total antioxidant status was calculated for samples from each study group. Before red wine consumption TAS levels were decreased in older volunteers compared to young volunteers (P < 0.001, Figure 4). Despite this difference between young and old volunteers consumption of red wine had the same effect within both the young and old group demonstrating a significant increase in total antioxidant status after red wine consumption, young with wine (P = 0.026) and older with wine periods (P = 0.01, Figure 4). These changes correspond to the changes in GSH and MDA with red wine consumption for both young and older groups. The total antioxidant status of the red wine consumed by all treatment subjects in this study contained 1.53 ± 0.027 mmol/L of antioxidant capacity (Figure 4).

There was no significant difference in both age (yrs) and BMI (kg/m2) between red wine and abstinence periods, for both young and older population groups (Table 2). Similarly there were no differences in serum glucose concentrations between pre and post samples for both young and older control and treatment groups (Table 2). Plasma lipid profiles for each study group were examined through the determination of plasma cholesterol, plasma triglycerides, plasma HDL-cholesterol and plasma LDL-cholesterol values. No statistical significance was found for any of the blood lipid profiles within each study group (Table 2).

**Table 2:** Anthropometric and blood lipid measurements

		**Young (18–30 yrs)**		**Older (≥ 50 yrs)**	
		**Abstain**	**Red wine**	**Abstain**	**Red Wine**
**Age (yrs)**		21.7 ± 0.3	21.3 ± 0.4	58.9 ± 2.3	57.2 ± 2.7
**BMI (kg/m**^2^**)**		24.7 ± 1.5	23.6 ± 1.2	32.5 ± 2.2	28.3 ± 2.2
**Cholesterol**	*Pre Sample*	4.3 ± 0.2	5.1 ± 0.5	5.9 ± 0.3	7.0 ± 0.2
**(mmol/L)**	*Post Sample*	3.2 ± 0.4	5.1 ± 0.1	5.8 ± 0.3	7.2 ± 0.2
**Triglycerides**	*Pre Sample*	1.7 ± 0.1	1.96 ± 0.2	2.3 ± 0.2	2.1 ± 0.1
**(mmol/L)**	*Post Sample*	1.8 ± 0.1	2.3 ± 0.1	2.5 ± 0.1	2.4 ± 0.2
**HDL-Cholesterol**	*Pre Sample*	31.3 ± 1.6	33.6 ± 2.4	46.3 ± 3.4	42.1 ± 2.4
**(mg/dL)**	*Post Sample*	37.1 ± 2.6	32.5 ± 2.4	44.3 ± 1.5	41.9 ± 1.9
**LDL-Cholesterol**	*Pre Sample*	137.5 ± 10.6	115.1 ± 8.2	194.8 ± 10.4	230.1 ± 11.5
**(mg/dL)**	*Post Sample*	108.0 ± 11.1	131.9 ± 8.8	185.9 ± 10.4	225.5 ± 12.8
**Glucose**	*Pre Sample*	5.8 ± 0.3	6.1 ± 0.4	6.6 ± 0.5	6.2 ± 0.3
**(mmol/L)**	*Post Sample*	5.8 ± 0.3	6.1 ± 0.5	6.4 ± 0.46	6.5 ± 0.5

Mean values for age and BMI of young (18–30 yrs) and older (≥ 50 yrs) subjects, together with serum glucose and plasma lipid profiles of pre and post samples for each study group. Values are mean ± SEM.

## Discussion

The results of these experiments show that moderate consumption of red wine significantly altered redox balance in the circulation of both young and old individuals. Red wine consumption increased serum antioxidant capacity and decreased concentrations of plasma malondialdehyde and whole blood glutathione in both age groups. In contrast, concentrations of malondialdehyde, glutathione, and total antioxidant capacity were significantly different between young and old control groups. Old subjects had greater concentrations of whole blood glutathione and lower concentrations of plasma malondialdehyde when compared to young subjects. Furthermore, total antioxidant capacity in serum was lower in the old group when compared to the young group. Red wine consumption did not significantly alter plasma lipid profiles and there were no significant differences between young and old individuals.

Oxidative stress is the consequence of an imbalance of oxidants and antioxidants. Studies show that a high consumption of antioxidants can decrease levels of oxidative stress and decrease the incidence of CVD [14]. In the current study TAS increased after red wine consumption these results strongly suggest that in the presence of red wine consumption, total antioxidant status has the ability to increase significantly. This increase was slightly more prominent in older individuals who increased their TAS 16% after consuming the red wine for two weeks compared to the younger individuals who increased 7%, despite the fact that young individuals had higher resting concentrations of total antioxidant status. Our results are also shared in a study by Fernandez-Pachon and colleagues [21], who eluded that antioxidant values determined before wine intake were statistically different from those measured 30 minutes after consumption. Our study however, extends beyond Fernandez-Pachon's by advocating that the consistent consumption of wine may offer the ability to significantly enhance total antioxidant status over a longer period. This sustained elevation of TAS further confirms that the lower incidence of CVD in populations who consume red wine on a regular basis [14] is due to an increase in circulating oxidative protection. In addition it also suggests that a lifetime of red wine consumption is not needed to achieve a sustained increase in circulating oxidative protection, two weeks is long enough to boost TAS.

To further determine the extent of positive effects associated with and increase in TAS we measured MDA, as a biomarker of lipid peroxidation and found that there were significant reductions in MDA after red wine consumption for both young and older volunteers. This suggests that the consumption of red wine was also effective in protecting circulating lipid systems from oxidative damage in young and older volunteers. Whereas, young and older groups experienced no changes in MDA values without red wine. These results indicate that MDA as a marker of circulating lipid oxidation was significantly reduced, thereby representing a significant reduction in lipid peroxidation in participants who consumed the red wine. Our results are complimented by studies published by Fuhrman [22], which show that the propensity of lipids to undergo peroxidation was reduced by 20% in correspondence with the consumption of 400 mL/day of red wine for 2 weeks. Moreover, our results suggest that the antioxidant effect of dietary red wine on plasma lipid peroxidation could be related to the elevation of polyphenol concentration in plasma. Interestingly, the young group had higher levels of MDA compared with the older group prior to red wine consumption. This was an unexpected result, as theoretically it is believed that oxidative damage such as lipid peroxidation increases with age [23].

In addition to measuring TAS and MDA in the current study the levels of whole blood GSH were measured and it was found that red wine consumption decreased GSH in young and older volunteers. However, there were no significant reductions in GSH levels in the absence of red wine. This would suggest, that a reduction in GSH might be due to it being down regulated as a result of the increased level antioxidants derived from red wine eliminating additional reactive oxygen species. In addition, it would appear that the level of protection conferred was greatest amongst younger participants which demonstrated a 55% reduction in GSH levels compared to older participants who had a 44% reduction in GSH, after both groups consumed red wine. It was expected and consequently demonstrated in this study, that the older population groups have a higher resting GSH content compared with their younger counterparts. This data may be interpreted with respect to the free-radical theory of ageing, in which endogenous oxidative damage occurs at higher frequencies with older age [24]. Data from Fenech et al [25] showed that the protective effects against DNA damage could not be readily explained by the phenolic content of the red wine, however, in a subsequent study by Greenrod et al [18] their data suggested that the non-alcoholic fraction of red wine protects DNA from oxidative damage, however, this effect is not solemnly explained by the antioxidant catechin.

No significant difference in age and BMI between young or old volunteers was observed before or after red wine consumption. This data indicates that potential changes in BMI had no influence on interpretation of the data. As the results of this present study indicate, no significant changes in plasma cholesterol, triglycerides, HDL-cholesterol, LDL-cholesterol and serum glucose were evidenced before and after red wine consumption (Table 2). Our data provides evidence that corresponds to research by Akcay and colleagues [26] whose findings suggest there is no significant difference in LDL blood levels with respect to the consumption of a cabernet sauvignon red wine.

From our data, relationships may be draw with respect to the consumption of red wine. We found that both young and older volunteers demonstrated significant decreases in GSH and MDA and this was proportional to the serum increases in total antioxidant status. This data signifies the relationship between a reduction in antioxidant levels and lipid oxidation and an increase in antioxidant status, with the consumption of 400 ml/day of red wine for 2 weeks. This finding is important with respect to the long-term implications of red wine consumption. It suggests that the consistent consumption of red wine may confer prolonged oxidative protection, as opposed to conflicting research, which suggests plasma polyphenols only peak at 3 hours post consumption [21]. Our study controlled for these factors, where participants abstained from consuming any alternative alcoholic sources, grapes or grape products during the course of the study, as well as all blood samples were taken after 12–14 hrs fasting. Our findings shed further light on the nature of the beneficial effects of red wine consumption and gives supporting evidence for the recommendation that red wine provides protective effects for CVD. Also, drinking pattern and not just the total amount of red wine consumed is important in the association between intake and protection.

## Conclusion

In conclusion red wine consumption decreases oxidative stress and enhances total antioxidant capacity in the circulation. This decrease in oxidative stress and increase in total antioxidant capacity in circulation is important as the opposite set of circumstances has been implicated in the pathogenesis of CVD. The results produced from this study suggest that the potent antioxidant properties provided by red wine and potential protection from developing CVD highlight the relationship between red wine consumption and health.

## Competing interests

The author(s) declare that they have no competing interests.

## Authors' contributions

MM carried out initial experimental procedures, data collection, preliminary data analysis and a 1st draft of the manuscript. LL assisted in the drafting of the final manuscript interpretation of data. PL conceived, designed and coordinated the study, and carried out secondary experimental procedures and data collection to complete the crossover period of the study, together with drafting the manuscript.
